# Comparison of Cycle Threshold and Clinical Status Among Different Age Groups of COVID-19 Cases

**DOI:** 10.7759/cureus.24194

**Published:** 2022-04-16

**Authors:** Baijayantimala Mishra, Jai Ranjan, Prashanth Purushotham, Punyatoya Kar, Poesy Payal, Swarnatrisha Saha, Vaishnavi Deshmukh, Sivasankar Das

**Affiliations:** 1 Department of Microbiology, All India Institute of Medical Sciences, Bhubaneswar, Bhubaneswar, IND

**Keywords:** clinical status, symptomatic, transmission, ct value, covid-19

## Abstract

Introduction

The COVID-19 pandemic has shaken the entire world ever since its emergence in March 2020. The disease manifestation of COVID-19 has been more severe, with a high degree of mortality in the elderly than in the young population. The cycle threshold (Ct ) value obtained in the real-time polymerase chain reaction (RT-PCR) has been used as the surrogate marker of viral load. Therefore, assessing Ct value and clinical status among different age groups with SARS-CoV-2 infection is required to understand the viral kinetics and to assess the transmission potential of that particular age group.

Purpose

The aim of this study was to compare the viral load and clinical status among different age groups with COVID-19 infection.

Methods and materials

A retrospective cross-sectional study was carried out to analyze the Ct values of SARS-CoV-2 positive samples reported from April 2020 till May 2021. The results of 13,820 RT-PCR (reverse transcriptase-polymerase chain reaction) positive samples were included for analysis of Ct values. Ct values of confirmatory genes were taken into consideration, and Ct values below 25, >25 to 30, and >30 were categorized as high, moderate, and low viral load, respectively. Age group was stratified into ≤18 years (young), 18-60 years (adult), and >60 years (elderly). The data were analyzed using SPSS Windows Version 25.0.

Results

The mean Ct values were 27.9, 26, and 26.2 in the young, adult, and elderly age groups, respectively. The mean Ct values of young patients were significantly higher as compared to adult and elderly patients (p<0.05). The percentage of high viral load (Ct<25) was found to be significantly higher in adults and elderly (44.6% & 43.7%) as compared to young (32.2%) (p<0.001). Majority of the COVID-19 positive cases younger than 18 years (75.9%) were asymptomatic as compared to 64.5% and 59.7% in the adult and elderly age groups, respectively.

Conclusion

This study observed a significantly high proportion of viral load in the adult and elderly population, which plays a substantial contribution to SARS-CoV-2 transmission, whereas the majority of the young population being asymptomatic plays a major role as silent transmitters. The study reemphasizes the need for strict adherence to COVID-appropriate behaviors.

## Introduction

The world has been reeling from the devastating effects of the COVID-19 pandemic since March 2020. As of August 28, 2021, it has resulted in about 4.4 million deaths worldwide [1]. The disease manifestation of COVID-19 has been more severe, with a high degree of mortality in the elderly than in the young population [2]. Thus, understanding the viral kinetics of SARS-CoV-2 among different age groups is of vital importance. This will aid in determining appropriate therapy and in assessing the risk of transmission. Asymptomatic individuals are thought to be significant contributors to transmission, though not to the extent of symptomatic individuals [3,4]. Only few studies have examined if the viral load in asymptomatic patients is less than that in symptomatic patients, which may correlate with less transmissibility. Children being more mobile carry more potential for virus transmission. This again makes it important to study the cycle threshold (Ct) value in different age groups along with any associated symptoms in order to determine the risk of COVID-19 transmission by different age groups.

Viral load determination in respiratory samples is difficult, and therefore some studies have correlated viral load to Ct value and have indicated that the lower the Ct value, the higher is the viral load or the infectiousness and severity of the disease [5]. Therefore, assessing Ct value and clinical status among different age groups with SARS-CoV-2 infection is required to devise treatment and assess the transmission potential of that particular age group. The purpose of this study was to determine the relationship between Ct values and clinical status among different age groups.

## Materials and methods

The study was conducted in the Department of Microbiology, All India Institute of Medical Sciences, Bhubaneswar, India, which is designated by the Indian Council of Medical Research (ICMR) as a COVID-19 reverse transcriptase-polymerase chain reaction (RT-PCR) laboratory. Samples from various districts of Odisha as well as suspected COVID-19 cases presenting to our hospital were received in our laboratory for COVID-19 testing.

 A retrospective cross-sectional study was conducted to assess and analyze the Ct values of COVID-19 positive samples reported during the period of April 2020 to May 2021 from our laboratory. Ct values of different samples were compared with different age groups as well as clinical status. A total of 13,820 COVID-19 RT-PCR positive samples were included in the study for analysis. Median Ct values of ORF/RdRp and N genes, i.e., confirmatory genes, were taken into consideration for comparison. Ct values below 25, >25 to 30, and >30 were categorized as high, moderate, and low viral load, respectively. Age group was stratified into ≤18 years (young), 18-60 years (adult), and >60 years (elderly). The patients were classified as symptomatic and asymptomatic based on history and data available from the ICMR Specimen Referral Form (SRF). The Institutional Ethics Committee of our hospital approved the study (IEC: T/IM-NF/Micro/21/81).

The data were analyzed using SPSS Windows Version 25.0 (IBM Corp., Armonk, NY, USA) and Microsoft Excel (Microsoft Office 2016, Microsoft Corporation, Redmond, WA, USA). The Shapiro-Wilk test was used to analyze for normality among the Ct values of different groups. Independent t-test and Mann-Whitney U test were used to compare data in case of normal and skewed data, respectively. A p-value of <0.05 was considered statistically significant.

## Results

**Figure 1:**
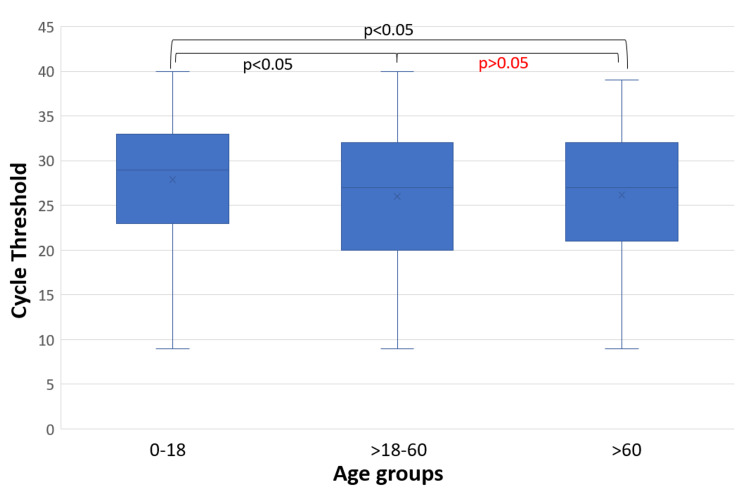
Comparison of Ct values across age groups

Ct values of a total of 1,011 young, 11,499 adult, and 1310 elderly COVID-19 positive patients were analyzed. The mean Ct values were 27.9, 26, and 26.2 in young, adult, and elderly age groups, respectively. The mean Ct values of young patients were significantly higher as compared to the adult and elderly patients (p<0.05) (Figure 1).

After comparing the viral load with each age group, a significant percentage of adults and elderly (44.6% and 43.7%, respectively) showed high viral load (Ct<25) compared to the young population (32.2%) (p<0.001) (Table 1).

**Table 1:** Viral load across different age groups

Age group	High viral load, n (%)	Moderate viral load, n (%)	Low viral load, n (%)
Young	325 (32.2)	250 (24.7)	436 (43.1)
Adult	5132 (44.6)	2461 (21.4)	3906 (34)
Elderly	573 (43.7)	291 (22.2)	446 (34.1)

**Figure 2:**
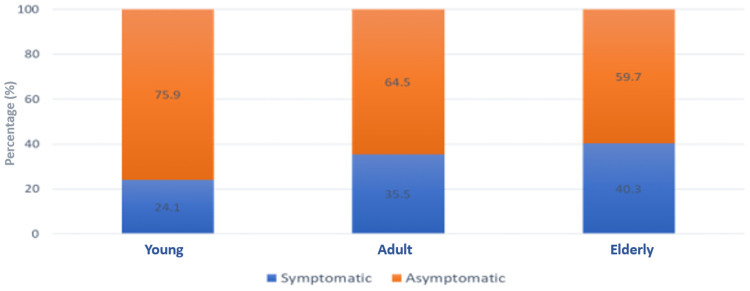
Comparison of clinical status among various age groups

Majority of the COVID-19 positive cases were younger than 18 years of age (75.9%) and were asymptomatic as compared to 64.5% and 59.7% of adult and elderly age groups, respectively, with a p-value of <0.05 (Figure 2).

**Figure 3:**
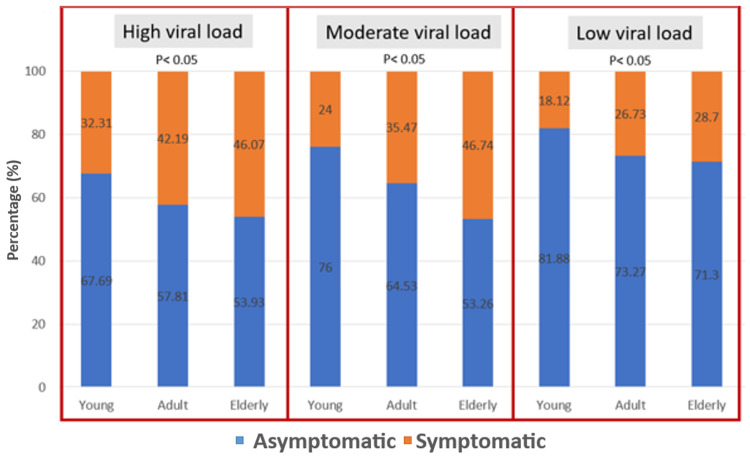
Comparison of clinical status among various age groups in high, medium, and low viral loads

When individual age groups were compared in terms of clinical features and individual categories of viral load (high, moderate, low), a significant percentage (67.6%) of young, asymptomatic population showed the presence of high viral load compared to asymptomatic adult and elderly groups in the same viral load category (57.8% and 53.9%, respectively). During the overall analysis, the percentage of asymptomatic individuals in each age group was higher in all the three categories of viral load: high, medium, and low viral loads (Table 2, Figure 3).

**Table 2:** Comparisons of clinical status and viral load in different age groups

Age group	Clinical status	High viral load, n (%)	Moderate viral load, n (%)	Low viral load, n (%)
Young	Symptomatic	105 (32.31)	60 (24)	79 (18.12)
	Asymptomatic	220 (67.69)	190 (76)	357 (81.88)
	Total (n=1,011)	325 (100)	250 (100)	436 (100)
Adults	Symptomatic	2,165 (42.19)	873 (35.47)	1,044 (26.73)
	Asymptomatic	2,967 (57.81)	1,588 (64.53)	2,862 (73.27)
	Total (n=11,499)	5,132 (100)	2,461 (100)	3,906 (100)
Elderly	Symptomatic	264 (46.07)	136 (46.74)	128 (28.7)
	Asymptomatic	309 (53.93)	155 (46.74)	318 (71.3)
	Total (n=1,310)	573 (100)	291 (100)	446 (100)

**Figure 4:**
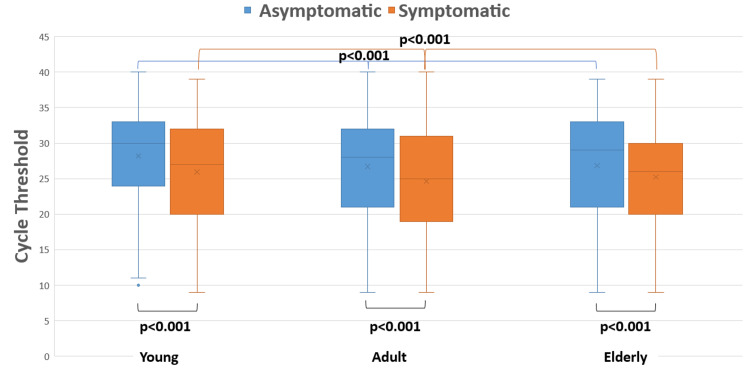
Comparison of Ct values in symptomatic and asymptomatic COVID-19 cases

Figure 4 shows the overall comparison of Ct values of symptomatic and asymptomatic patients among different age groups. The median (IQR) for asymptomatic patients in young, adult, and elderly was 30 (24-33), 28 (21-32), and 29 (21-33), and for symptomatic patients, it was 23 (20-32), 25 (19-31), and 26 (20-30), respectively. Among each age group, symptomatic Ct values were found to be lower than that of asymptomatic patients and all were statistically significant (p<0.001).

## Discussion

A significantly higher proportion of adults and elderly were found to have a higher viral load compared to the young individuals in our study. This could be a reason for more adults and elderly being severely affected by COVID-19 and having higher mortality rates. Immunosenescence, changes in T-cell diversity, inflammation [6], and the presence of more comorbidities among the elderly [7] could be the reasons behind severe COVID-19 among them.

The gold standard for diagnosis of COVID-19 infection is real-time RT-PCR [8]. It utilizes primer and probes, and the resulting fluorescence is detected. Ct value is the number of the PCR cycle at which the fluorescence signal crosses the threshold value, thus labelling a particular sample as positive or negative. Ct values are a surrogate marker of viral load and are inversely proportional to it. A lower Ct value signifies a higher viral load and vice versa [5].

In a study by Wright et al., lower Ct values are associated with severe disease and poor prognosis. This highlights that Ct values have an inverse association with clinical severity [9]. Undurraga et al. in their study from Chile have also found COVID-19 to disproportionately affect the elderly [10]. In our study, in an overall manner, a significant proportion of adults and elderly had a lower Ct value (Figure 1, Table 1), which corroborates the finding that a severe COVID-19 is predominantly found among these two age groups because of high viral load and increased viral shedding in them [11]. The presence of higher viral load, presence of co-morbidities, and a decreased immune response can, in turn, lead to a higher case fatality rate among the elderly [11]. This supports the findings of O’Driscoll et al., who in their study had found a strong correlation between age and mortality due to COVID-19 [12].

However, studies by Jacot et al. [13] and Mahallawi et al. [14] did not find any substantial association between age groups and higher viral loads (assessed by Ct values). This could be due to the fact that high viral load is one of the several factors that is responsible for causing severe COVID-19 disease.

Ct values can be considered an indicator of virus transmissibility, with lower Ct values indicating a higher viral load [15] and, in turn, increased viral shedding. Hence, Ct values can be utilized to assess the viral load especially among asymptomatic individuals to ascertain the transmission potential. In our study, we found that the proportion of asymptomatic individuals was higher compared to the symptomatic ones across all age groups (Table 2, Figure 3). Overall, a significant proportion (75.9%) of young individuals were found to be asymptomatic. This is in concordance with the study by Al-Qahtani et al. [16], who also found asymptomatic patients to be in larger numbers compared to individuals with symptoms of COVID-19. Another major finding in our study was that a higher percentage of young asymptomatic people (67.6%) were having high viral load compared to the other two groups with the same viral load (Ct<25) (Table 2, Figure 3). These individuals not only pose a risk to the general population but can also act as silent transmitters of COVID-19.

On the other hand, the median Ct values of symptomatic patients were found to be lower with respect to the asymptomatic individuals in our study across all age groups (Figure 4). This signifies that symptomatic individuals were found to harbor more viruses. This is in corroboration with the studies by McEllistrem et al. [17] and Strutner et al. [18], wherein symptomatic individuals were found to have higher viral load.

The findings in our study may be an indication that asymptomatic COVID-19 positive individuals can act as a reservoir of infection and contribute substantially to virus transmission and surge of the COVID-19. This is more imminent when asymptomatic cases are harboring high viral load. The high positivity in the asymptomatic individuals also reiterates the importance of contact tracing. In the current scenario, with more coverage of COVID-19 vaccination, it is expected to get a greater number of asymptomatic COVID-19 positive individuals in coming days, which might play a role in facilitating the disease transmission.

The major limitation of the present study is that it is a single-center study. Multi-centric study results will give a better understanding of the correlation between Ct values and clinical status. In addition, further studies may be undertaken to evaluate Ct values among people with severe symptoms, i.e., hospitalization or ICU admission, and asymptomatic individuals would also provide an insight into the association between the two. The present study predominantly looked at the correlation of age and clinical features in terms of symptomatic and asymptomatic with Ct values of the positive samples. However, as this is a retrospective study, other patient characteristics such as comorbidities and vaccination status could not be analyzed.

## Conclusions

The present study observed that a significantly high proportion of adult and elderly SARS CoV-2 positive patients are symptomatic and have high viral load. Thus, symptomatic adult and elderly patients play a substantial role in SARS-CoV-2 transmission. On the other hand, young people being mostly asymptomatic even with high viral load may play a significant role in SARS-CoV-2 transmission in a cryptic manner. This study reemphasizes the need for strict adherence to COVID appropriate behaviors for all, effective contact tracing, and early testing in all symptomatic individuals in order to prevent further surges of COVID-19.
